# Geomagnetic Field (Gmf) and Plant Evolution: Investigating the Effects of Gmf Reversal on *Arabidopsis thaliana *Development and Gene Expression

**DOI:** 10.3791/53286

**Published:** 2015-11-30

**Authors:** Cinzia M. Bertea, Ravishankar Narayana, Chiara Agliassa, Christopher T. Rodgers, Massimo E. Maffei

**Affiliations:** ^1^Department of Life Sciences and Systems Biology, University of Turin; ^2^Radcliffe Department of Medicine, University of Oxford, John Radcliffe Hospital

**Keywords:** Developmental Biology, Issue 105, Geomagnetic field, *Arabidopsis thaliana*, plant development, gene expression, antioxidant genes, magnetic field reversal, triaxial Helmholtz coils

## Abstract

One of the most stimulating observations in plant evolution is a correlation between the occurrence of geomagnetic field (GMF) reversals (or excursions) and the moment of the radiation of Angiosperms. This led to the hypothesis that alterations in GMF polarity may play a role in plant evolution. Here, we describe a method to test this hypothesis by exposing *Arabidopsis thaliana* to artificially reversed GMF conditions. We used a three-axis magnetometer and the collected data were used to calculate the magnitude of the GMF. Three DC power supplies were connected to three Helmholtz coil pairs and were controlled by a computer to alter the GMF conditions. Plants grown in Petri plates were exposed to both normal and reversed GMF conditions. Sham exposure experiments were also performed. Exposed plants were photographed during the experiment and images were analyzed to calculate root length and leaf areas. Arabidopsis total RNA was extracted and Quantitative Real Time-PCR (qPCR) analyses were performed on gene expression of *CRUCIFERIN 3* (*CRU3*), *copper transport protein1* (*COTP1*), *Redox Responsive Transcription Factor1* (*RRTF1*), *Fe Superoxide Dismutase 1*, (*FSD1*), *Catalase3* (*CAT3*), *Thylakoidal Ascorbate Peroxidase* (*TAPX*), a cytosolic *Ascorbate Peroxidase1* (*APX1*), and *NADPH/respiratory burst oxidase protein D* (*RbohD*). Four different reference genes were analysed to normalize the results of the qPCR. The best of the four genes was selected and the most stable gene for normalization was used. Our data show for the first time that reversing the GMF polarity using triaxial coils has significant effects on plant growth and gene expression. This supports the hypothesis that GMF reversal contributes to inducing changes in plant development that might justify a higher selective pressure, eventually leading to plant evolution.

**Figure:**
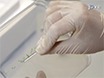


## Introduction

The Earth’s magnetic field (or equivalently the geomagnetic field, GMF) is an inescapable environmental factor for all organisms living on the planet, including plants. The GMF has always been a natural feature of the Earth, so during evolution, all living organisms experienced its action. An increasing body of evidence shows that the GMF is able to influence many biological processes ^1^. The GMF is not uniform and there are significant local differences in its magnitude and direction at the surface of the Earth. The GMF at the Earth's surface shows a broad range of magnitudes, ranging from less than 30 µT to almost 70 µT. The GMF protects the Earth and its biosphere from the lethal effects of solar wind by deflecting most of its charged particles through the magnetosphere^2^.

Plants respond to environmental stimuli; and classical responses to abiotic factors such as light and gravity have been thoroughly described by defining the so-called phototropic and gravitropic responses. Very little, or nothing, is known on the mechanisms of perception and responses of plants to magnetic fields, despite the plethora of papers published on this topic and recently reviewed ^1^. Unlike the gravitational field, the GMF changed consistently during plant evolution thereby representing an important abiotic stress factor that has been recently considered a potential driving force eventually contributing to plant diversification and speciation ^2^. Geomagnetic reversals (or excursions) are changes in polarity of the GMF. During Earth’s life-history, GMF reversals occurred several times. These exposed the planet to periods of reduced GMF strength during every polarity transition. Some authors have hypothesized that these transitions periods of low GMF strength might have allowed ionizing radiation from the solar wind to reach the Earth’s surface, thereby inducing a consistent stress to living organisms, which could have been strong enough to induce gene alterations eventually leading to plant evolution ^2^.

A detailed analysis of experiments describing the effects of magnetic fields on plants shows a large number of conflicting reports, characterized by a dearth of plausible biophysical interaction mechanisms. Many experiments are simply unrealistic, while others lack a testable hypothesis and, ultimately, are unconvincing ^3^. Over the past years, the progress and status of research on the effect of magnetic fields on plant has been reviewed ^2,4-11^. Recently, the effect of both low and high magnetic field has been thoroughly discussed ^1^, with a particular focus on the involvement of GMF reversal events on plant evolution ^2^.

The most direct means to substantiate the hypothesis that GMF reversals affect plant evolution is to synthesize a GMF reversal in the laboratory by testing the response of plants to normal and reversed magnetic field conditions. To test the hypothesis, we therefore built a triaxial octagonal Helmholtz coil-pairs magnetic field compensation system (triaxial coils), which is able to accurately reverse the normal GMF conditions.

We used *Arabidopsis thaliana* as a model plant and we tested the effect of reversed GMF on gene expression of some important genes: *CRUCIFERIN 3* (*CRU3*), that encodes a 12S seed storage protein that is tyrosine-phosphorylated and its phosphorylation state is modulated in response to ABA in *Arabidopsis thaliana* seeds ^12,13^; the *Copper Transport Protein1* (*COTP1*), that encodes a heavy metal transport/detoxification superfamily protein with the predominant function in soil Cu acquisition and pollen development ^14^; and *Redox Responsive Transcription Factor1* (*RRTF1*), that encodes a member of the ERF (ethylene response factor) subfamily B-3 of ERF/AP2 transcription factor family that contains one AP2 domain that facilitate the synergistic co-activation of gene expression pathways and confer cross tolerance to abiotic and biotic stresses ^15^.

Moreover we also analyzed five genes involved in oxidative stress responses: *Fe Superoxide Dismutase1*, (*FSD1*), that encodes a cytoplasmic enzyme that enzymatically and rapidly converts the superoxide anion (O_2_^-^) and water (H_2_O) to hydrogen peroxide (H_2_O_2_) and molecular oxygen (O_2_) ^16^; *Catalase3* (*CAT3*), that that encodes and enzyme that catalyzes the breakdown of H_2_O_2 _into water and oxygen ^17,18^; *Thylakoidal Ascorbate Peroxidase* (*TAPX*), that encodes a chloroplastic thylakoid peroxidase that scavenges H_2_O_2 _^19^;*Ascorbate Peroxidase1* (*APX1*), that encodes a cytosolic peroxidase that scavenges H_2_O_2 _and represents one of the potential targets of post-translational modifications mediated by NO-derived molecules ^20^; and NADPH-*Respiratory burst oxidase protein D* (*RbohD*) that encodes an enzyme that generates O_2_^- ^and plays pivotal roles in regulating growth, development and stress responses in Arabidopsis ^21^.

Our field-reversal methodology provides the first evidence that GMF reversal can induce a significant change in the morphology and gene expression of *A. thaliana* roots and shoots. This protocol provides an innovative way to evaluate the effect of GMF reversal on plant morphology and gene expression and can be used to assess the potential effect of GMF reversal on other aspects of plant behavior, and thereby guide discussion of the role of GMF reversal on plant evolution.

## Protocol

### 1. Setting of the Triaxial Coils

NOTE: **Figure 1** shows the triaxial coils used to reverse the GMF.

1. Turn on the three-axis magnetometer, whose probe is inserted in the triaxial coils.
2. Turn on the computer and launch the magnetometer software that allows data to be collected from three-axis magnetometer.
3. Use the component values reported by the magnetometer to calculate the magnitude of the GMF. For instance, with magnetometer values: Bx = 6.39 µT, By = 36.08 µT, Bz = 20.40 µT calculate a field strength of 41.94 µT by using the following equation: B = B_GMF_ + B_additional_, where B_additional_ = (Bx^2^ + By^2^ + Bz^2^)^½^ (*i.e.,* 41.9 µT in the example.)
4. Turn on the three DC power supplies (dual range: 0-8V/5A and 0-20V/2.5A, 50W) each one connected to three couples of Helmholtz coils and connected to a computer via a GPIB connection (**Figure 1B**).
5. Set voltages of the power supplies to generate the desired magnetic field with a reversed magnetic field vector. For instance, with B_GMF _as in step 1.3 and with the coil size of the instrumentation described here, set the voltages to V_x_ =0.00 V, V_y_ = 30.52 V, V_z_ = 0.00 V in order to generate a new resultant B = B_GMF_ + B_triaxial coils_ = (6.38, -36.08, 20.39) µT. *i.e.,* a new field with the same magnitude as B_GMF_ but pointing to a different direction.
6. Verify the new field with the magnetometer software by using the procedure described in 1.3.
7. Expose plants to both normal and reversed GMF conditions by using Petri plates as described in section 2.
8. Perform sham exposure experiments by keeping the magnitude of the field equal to | B_GMF_| and keeping**the vertical component of the field equal to that of the GMF but altering the direction (*i.e.,* “North, East or West”) of the horizontal component of the field with equal currents in the triaxial coils compared to the field reversal condition. Do this by altering the voltage of the coils as described in 1.5. Note: This sham exposure rules out potential subtle heating or vibrational effects from either the coils themselves or from the electronics used to control the coils.
9. Run double-blind experiments by applying field conditions blinded from the personnel performing the remainder of the experiments and/or interpreting the data.

### 2. Preparation of Plant Materials and Conditions for Plant Growth

1. Use seeds of *Arabidopsis thaliana*, ecotype Columbia 0 (Col 0), place then into a 1.5 ml tube and surface sterilize by treatment with a 5% (w/v) calcium hypochlorite solution and 0.02% (v/v) Triton X-100 in 80% ethanol (EtOH), for 10-12 min at 25-28 °C, with continuous shaking. Then rinse twice with 80% EtOH, wash with 100% EtOH and finally rinse with sterile distilled water.
2. Prepare 1 L of Murashige and Skoog ^22^ (MS) modified medium by adding: 2.297 g of MS (0.5 x MS Basal Salt Mixture), 10 g Sucrose, deionized water up to 1 L, pH 5.8-6.0 adjusted with KOH. Add 16 g of agar and autoclave for 20 min, 120 °C.
3. Before solidification, pour 80 ml of medium into each (120 x 120 mm^2^) square Petri plates. Sow thirty sterile seeds on plate, and then seal plates with a waxy film.
4. Vernalize the plates horizontally in darkness at 4 °C for 2 days to potentiate and synchronize germination, and then expose Petri plates to either normal or reversed GMF.
5. Expose seeds in a climate controlled environment at 22 °C in a vertical position in parallel experiments both inside the triaxial coils and outside the triaxial coils under a photoperiod schedule of 8 hr darkness and 16 hr light, using Sodium vapor lamps (220 x 10^-6^ E m^-2^s^-1^). Use a blue gelatin film for spotlight to reduce the red component of lamps.
6. Expose plants for 10 days before RNA extraction to both normal (control) and reversal (treatment) GMF conditions.
7. After exposure, take pictures of Petri dishes.
8. Use the ImageJ software to calculate root length and leaf areas.
  1. Briefly, measure the side of the Petri plate, then open the image of the Petri plate and by using the “straight” line option to draw a line that exactly crosses the plate side.
  2. In the “analyze” menu select “set scale” and insert the actual distance in the “known distance” box (*e.g.*, 120 mm), then insert the unit of length (mm); finally click the “global” option to make settings available for all measurements.
  3. For root length, follow carefully the shape of the root by using the freehand tool. Measure the length by using the “measure” option in the “analyze” menu. Continue to measure all roots in the picture and save the file for further statistical analyses.
  4. For leaf area, from the “image” menu use the adjust option and then “color threshold”. Select the individual leaf and in the “analyze” menu select “analyze particles”. Save the individual measurements for statistical analyses.

### 3. *Arabidopsis* Total RNA Extraction, Quantitative Real Time-PCR (qPCR) Reaction Conditions and Primers for *Arabidopsis*

1. Collect separately 30 shoots and 30 roots and immediately freeze in liquid nitrogen. Then grind in liquid nitrogen with mortar and pestle.
2. Isolate total RNA using a purification kit and RNase-Free DNase treatment kit by using manufacturer’s instructions.
3. Check sample quality and quantity by using an RNA nano kit and capillary gel electrophoresis according to manufacturer’s instructions. Confirm the quantification of RNA spectrophotometrically.
4. Use 2 µg of total RNA and random primers using a cDNA Reverse Transcription Kit to obtain first strand cDNAs according to the manufacturer’s recommendations.
5. Perform all experiments on a Real-Time System using SYBR green I with ROX as an internal loading standard.
6. Perform the reaction with 25 µl of mixture consisting of 12.5 µl of 2x SYBR Green qPCR Master Mix, 0.5 µl of cDNA and 100 nM primers. Use the primers listed in **Table 1**. Include in controls non-RT controls (using total RNA without reverse transcription to monitor for genomic DNA contamination) and non-template controls (water instead of template).
7. Calculate primer efficiency for all primers pairs using the standard curve method ^23^.
8. Use the following PCR conditions: *CRU3*, *COTP1*, *RRTF1*. 10 min at 95 °C, 40 cycles of 15 s at 95 °C, 30 sec at 58 °C, and 30 sec at 72 °C; *UBP6*, *eEF1Balpha2*, *ACT1*, *GAPC2*, *CAT3*, *TAPX*, *APX1*, *RbohD*, *FeSOD1* 10 min at 95 °C, 40 cycles of 15 s at 95 °C, 20 sec at 57 °C, and 30 sec at 72 °C.
9. Read fluorescence following each annealing and extension phase. For all runs, perform a melting curve analysis from 55 to 95 °C by including the dissociation segment in the thermal profile. Use the dissociation curve screen, accessed through the results tab to view the dissociation profile (plot of the fluorescence as a function of temperature). Ensure that the dataset collected during the dissociation segment of the experiment is selected for analysis using the Analysis/Setup screen.
10. Determine the linear range of template concentration to threshold cycle value (Ct value) by performing a tenfold dilution series (1- to 10^3^-fold) using cDNA from three independent RNA extractions analyzed in three technical replicates ^24,25^.
11. Analyze all amplification plots with the Real-Time PCR instrument software to obtain Ct values. Calibrate and normalize relative RNA levels with the level of the best housekeeping genes as follows: 3.11.1) Access the Amplification Plots screen through the Results tab, select the ramp or plateau for which data should be analyzed using the Analysis Selection/Setup screen, and then select the dRn (baseline-corrected normalized fluorescence) from the Fluorescence menu on the command panel. Access the Plate Sample Values screen through the Results tab to display Ct values for the sampled wells.
12. Use four different reference genes [*e.g.*,* cytoplasmic glyceraldehyde-3-phosphate dehydrogenase, (GAPC2), ubiquitin specific protease 6 (UBP6), Actin1 (ACT1) and the elongation factor 1B alpha-subunit 2 (eEF1Balpha2)*] to normalize the results of the real time PCR. Select the top ranked gene using an analysis software ^26^; and use the most stable gene for normalization.
  1. Briefly, organize the input data on an Excel sheet with the first column containing the gene names and first row containing the sample names. Then select the analysis software from the menu-bar. Use the dialog box to select the input data.
  2. Next, check the fields Sample names, gene names and simple output only. Click on Go button to perform the analysis. Select the top ranked gene (which has the smallest stability value) as candidate gene most stably expressed.
13. Plot data by showing the differential fold change expression in both shoots and roots.

### 4. Statistical Analyses

1. Express data as mean values ± standard error. Compare the control and the treatment groups by performing analysis of variance (ANOVA) and Tukey’s test with Bonferroni and Dunn-Sidak Adjusted Probability test (0.95% confidence).

## Representative Results

The aim of this protocol is to provide a method to assess whether reversal of the geomagnetic field (GMF) may affect plant development and gene expression of *Arabidopsis thaliana* ecotype Col 0. Triaxial coils as shown in **Figure 1A** are used to reverse the GMF when set with the appropriate drive voltages (**Figure 1B**), obtained as described in step 1.5 in the protocol. The dimensions of the triaxial coils are ~2 x 2 x 2 m^3^, which allowed sufficient space with reversed GMF conditions to host several Petri plates. Controls were grown in the same environmental conditions and at normal GMF values. After 10 days of exposure to normal and reversed GMF conditions, the phenotype of plants showed evident morphological alterations. As shown in **Figure 2**, control plants (*i.e.*, grown in normal GMF conditions) showed root lengths with significantly (Dunn-Sidak and Bonferroni Adjusted Prob <0.001; Student’s t = 10.68, df = 31) higher values (29.41 mm; SEM = 1.04; N = 32) with respect to plants exposed to reversed GMF (17.53 mm; SEM = 0.58; N = 36). In GMF-reversed plants, the morphology of shoots was also altered by showing a reduced development of leaflet expansion. Plants exposed to normal conditions showed an average leaf area of 4.95 mm^2^ (SEM 0.025, N = 54), whereas plants exposed to reversed GMF conditions showed significantly (Dunn-Sidak and Bonferroni Adjusted Prob = <0.001; student’s t = 31.32, df = 53) lower leaf area values (3.71 mm^2^; SEM = 0.032; N = 54). Therefore, exposure of Arabidopsis to reversed GMF conditions induced a reduction in both root length and leaf area.

Leaf expansion and root growth are dependent on both the division and the elongation of cells ^27^. Therefore, plant development, productivity and overall fitness are dependent on an optimal shoot- and root-system architecture ^28^. The reduced root length and leaf size of plants exposed to reversed GMF conditions indicate the presence of a sensing system able to not only perceive variations in magnetic field intensity, but also to respond to changes in the magnetic field “direction” compared to gravity. The hypothesis that GMF reversal may affect plant growth finds compelling evidence in our experiments, which demonstrate that GMF reversal conditions can significantly affect plant development.

The morphological changes were also accompanied by changes in gene expression. Among housekeeping genes, the most stable gene was the *elongationfactor1B alpha-subunit 2*. The first group of genes (*CRU3*, *COTP1*, *RRTF1*) showed a dramatic alteration in the gene expression (**Figure 3**). Shoot expression of all three genes was significantly increased (P <0.05) by about 2.5-fold in plants exposed to reserved GMF conditions. Root expression of *CRU3* was upregulated in the roots in plants exposed to normal GMF conditions, but was significantly (P <0.05) downregulated in reversed GMF conditions. The opposite was found for *COTP1 *and *RRTF1*, which were downregulated in normal conditions and upregulated in the presence of GMF reversal (**Figure 3**).

Cruciferin (a 12 S globulin) is the most abundant storage protein in the seeds of *A. thaliana* and other crucifers and is synthesized as a precursor in the rough endoplasmic reticulum. It is then transported to the protein storage vacuoles ^13^. Seedling germination requires the breakdown of cruciferin, which is used as an initial source of nitrogen. Down-regulation of cruciferin degradation reduces embryos development by impairing cell structures or cell components development ^29,30^. Our results show that upregulation of *CRU3* correlates with a lower leaf expansion and a reduced root length, thus indicating that this gene in sensitive to GMF reversal and that its overexpression may contribute to the reduction of plant development. Moreover, GMF reversal induces a significant downregulation of *CRU3* in roots, which correlates with a reduced root length. Copper is an essential cofactor for key processes in plants, but it exerts harmful effects when in excess; thus, overexpressing copper transport compromises plant growth. The effect of GMF reversal was a significant overexpression of *COTP1* in both shoots and roots, thus explaining the reduced plant growth. Ion stress impairs chloroplast metabolism, which is tightly linked to the redox state of the cell. In Arabidopsis the transcription factor RRTF1 is important for the expression of genes associated to the ability to adjust to redox changes ^31^. Therefore, when plants are exposed to external stimuli able to alter their physiological and developmental programs an overexpression of this important transcription factor is expected. Reversal of the GMF induced a significant overexpression of *RRTF1* in both shoots and roots, thus indicating higher oxidative stress responses of plants to reversed GMF conditions.

Interesting results are obtained by analyzing the five genes involved in oxidative stress. In general, all genes extracted and analyzed in shoots did not show significant differences (P >0.05) when plants were grown in normal or reversed GMF conditions (**Figure 4** and **Figure 5**). However, a significant down-regulation was always observed in roots of plants exposed to reversed GMF conditions. In particular, *CAT3* showed the highest downregulation (**Figure 5**), followed in order of downregulation by *APX1*, *FSD1*, *RBOHD* and *TAPX* (**Figure 4**).

Cross tolerance to abiotic and biotic stress is provided by the activation of different genes involved in several biochemical pathways.* RRTF1* transcription factor facilitates the synergistic co-activation of gene expression of these pathways ^15,31^, and can be potentially involved in oxidative stress ^32^. Therefore, upregulation of *RRTF1* is expected when oxygen scavenging is reduced. Downregulation of root scavenging enzymes correlates with the upregulation of *RRTF1*, which acts in response to increased oxidative stress. The dramatic root downregulation of *CAT3*, *APX1* and *TAPX* indicates the reduced ability of root cells to scavenge H_2_O_2_, which is accompanied by the reduced ability to dismutate the superoxide anion by downregulation of *FSD1*. The oxidative stress responses is higher in roots, which appear to be the main site of reversed GMF perception.


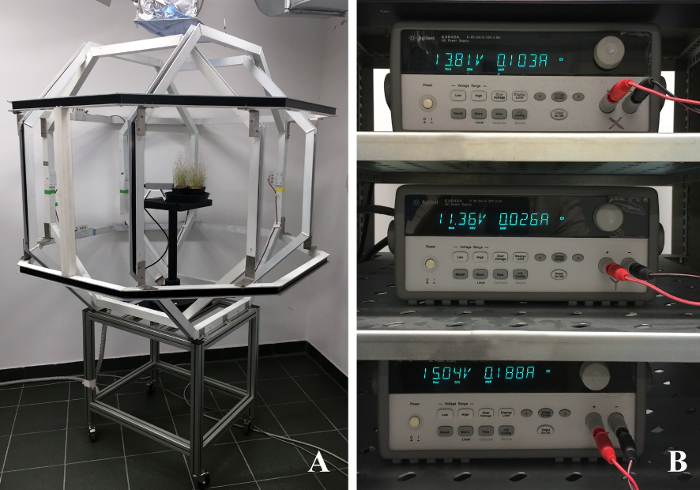
**Figure 1. Geomagnetic field Compensation system**. (**A**) triaxial coils (comprising a pair of octagonal coils for each of three perpendicular axes) used to reverse the geomagnetic field vector. (**B**) A computer-controlled power supply is connected to each pair of Helmholtz coils. (Voltages in these figures are arbitrary) Please click here to view a larger version of this figure.


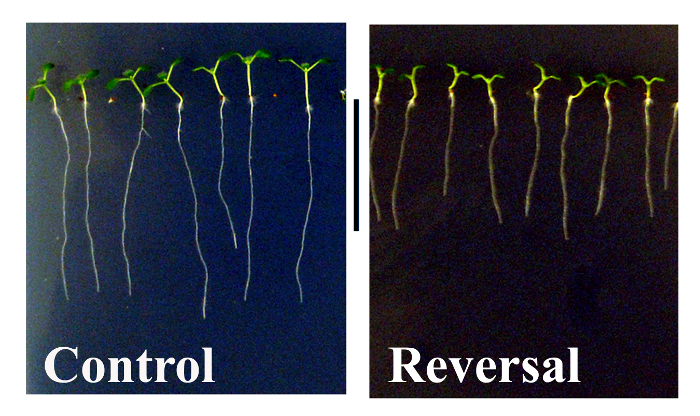
**Figure 2. Effects of geomagnetic field reversal on Arabidopsis morphology**. After ten days of exposure, control plants (*i.e.*, those exposed to normal GMF conditions) show a significantly greater root length and more expanded leaflets compared to plants that were exposed to reversed GMF conditions. Metric bar = 18 mm. Please click here to view a larger version of this figure.


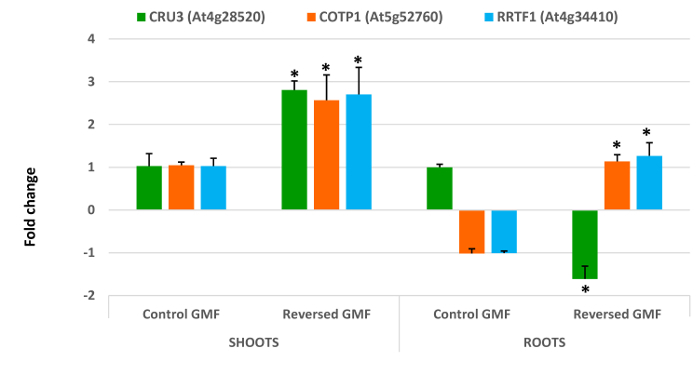
**Figure 3. Effects of geomagnetic field reversal on Arabidopsis gene expression**. After ten days of exposure, total RNA of control and treated plants was extracted and analysed by Real-Time PCR for expression analysis. The effect of reversal of the GMF was to induce a drastic change in the gene expression of all genes that were tested. *CRU3*, *Cruciferin 3*; *COTP1*, *Copper Transport Protein1*; *RRTF1*, *Redox Responsive Transcription Factor1*. Bars indicate standard error; asterisks indicates significant (P <0.05) differences between plants exposed to reversed and normal GMF conditions. Please click here to view a larger version of this figure.


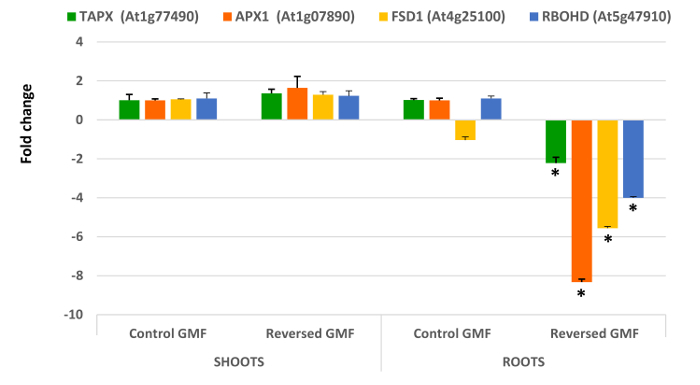
**Figure 4. Effects of geomagnetic field reversal on Arabidopsis antioxidant-related gene expression**. After ten days of exposure, total RNA of control and treated plants is isolated and employed for gene expression analysis using Real-Time PCR. The effect of reversal of the GMF was to induce no significant changes in shoot gene expression; however, a drastic downregulation was observed in root gene expression of plants grown under reversed GMF conditions. *TAPX*, *Thylakoidal Ascorbate Peroxidase*; *APX1*, *Ascorbate Peroxidase1*; *FSD1*, *Fe Superoxide Dismutase1*; *RbohD*, *NADPH/Respiratory burst oxidase protein D*. Bars indicate standard error; asterisks indicates significant (P <0.05) differences between plants exposed to reversed and normal GMF conditions. Please click here to view a larger version of this figure.


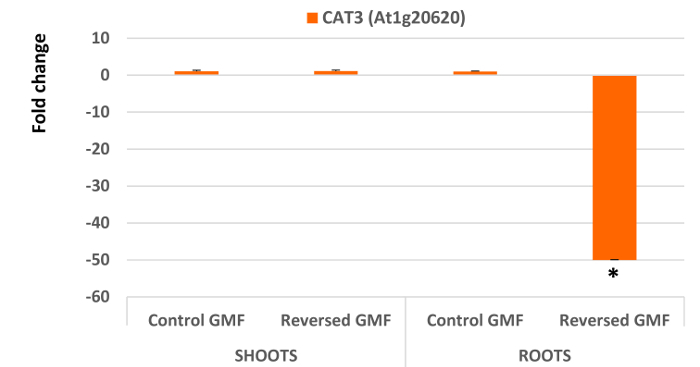
**Figure 5. Effects of the geomagnetic field reversal on *Arabidopsis *Catalase 3 (CAT3) gene expression**. After ten days of exposure, total RNA of control and treated plants is isolated and employed for gene expression analysis using Real-Time PCR. The effect of reversal of the GMF was to induce no significant changes in shoot gene expression; however, a drastic downregulation was observed in root gene expression of plants grown under reversed GMF conditions. Bars indicate standard error; asterisks indicates significant (P <0.05) differences between plants exposed to reversed and normal GMF conditions. Please click here to view a larger version of this figure.

## Discussion

We recently showed that an amazing correlation exists between GMF reversals and the time when diversion of most of the familial Angiosperm lineages occurred ^2^. However, despite the stimulating hypotheses and the plethora of studies on the effect of varied GMF intensities, the assumption that GMF reversal may itself induce significant changes in plant gene expression and morphology has never been demonstrated. Here we show for the first time, a method that uses a triaxial octagonal Helmholtz coil to reverse GMF in our laboratory, and that the reversal of the ambient magnetic field can cause phenotypic changes and modulation of gene expression in plants.

In order to obtain GMF reversal (or modification) over a sufficient volume for the plant growth experiments (2 x 2 x 2 m^3^), we built an octagonal Helmholtz coil system. This system is not commercially available (usually Helmholtz coils are ring-shaped and smaller) and the costs for construction were considerable. Importantly, this system delivers robust field modification, with exceptional time-stability and homogeneity in the modified magnetic fields.

The system is designed and built to reduce the value of the GMF to a thousandth of the normal conditions or to reverse any of the three dimensions of the magnetic field. However, the design of the coils does not allow to generate a high magnetic field strength. Therefore, this instrument in the present form is not suitable for experiments designed to evaluate the effect of high magnetic field strength on plants or other organisms.

In laboratory, alteration of the GMF similar to those described in this method has been obtained by different methods, including shielding by surrounding the experimental zone by ferromagnetic metal plates with high magnetic permeability, which deviate magnetic fields and concentrate them within the metal itself. The advantage of using Helmholtz coils is that the system allows plants to be exposed to more natural conditions (light, air circulation, *etc.*), thus making it ideal not only for* in vitro* studies (as with the use of Petri dishes) but also for *in vivo* plant growth and development experiments. The dimensions of our system create a space that allows a suppression up to <1/1,000th of the natural GMF throughout a 25 x 25 x 25 cm^3^ spherical volume (see **Figure 1A**), thus allowing to host several Petri plates or some small pots for plant growth.

The method presented here has been applied to plant biology studies; however, the system allows a wide range of experimentation, including virology and microbiology, as well as studies on nematodes (*e.g.*, *Caenorhabditis elegans*), arthropods and small animals (including mice and rats). Therefore, tests of the hypothesis that reversal of the GMF is able to induce morphological and transcriptional changes could also be extended to many other living systems, perhaps ultimately even to human cells.

The GMF is constantly changing and fluctuating. Therefore, in our experiments one major challenge is to provide a constant compensation of the GMF in order to obtain the desired new GMF values. This can only be achieved by a continuous control of magnetic fields values through reading of magnetometer values and voltage compensation. Therefore, the system can compensate the slowly varying part of the GMF but it does nothing for higher frequency fluctuations.

In conclusion, the use of triaxial coils to reverse the GMF vector was instrumental to demonstrate that this reversal of the GMF vector is able to induce plant morphological changes and differential gene expression. The results obtained with the presented method provide a compelling evidence in support of the hypothesis that the GMF reversals might have been one of the driving forces for plant evolution over geological timescales ^2^.

## Disclosures

The authors have nothing to disclose.
